# Assessment of medical waste management practices and implications for antimicrobial resistance risk in urban Nigeria

**DOI:** 10.1186/s12889-026-28840-w

**Published:** 2026-08-14

**Authors:** Gbolahan Afeez Yusuf, Oluremi Nurudeen Olaleye

**Affiliations:** 1 Crown University International, Delaware, USA; 2 Brandenburg University of Technology, Cottbus-Senftenberg, Germany; 3 Lagos State University of Science & Technology, Ikorodu, Lagos State, Nigeria

**Keywords:** Antimicrobial resistance, Healthcare waste, Pharmaceutical waste, Environmental health, Lagos, Nigeria

## Abstract

Antimicrobial resistance (AMR) is a major global public health threat. Medical waste management is a potentially important but underexplored pathway for environmental dissemination of antimicrobial residues and resistant microorganisms, particularly in low- and middle-income countries. A concurrent mixed-methods study was conducted in seven healthcare facilities in Lagos State, Nigeria. Quantitative data were collected through structured questionnaires administered to 336 healthcare workers and facility assessments. Qualitative data were obtained through semi-structured interviews with 49 key informants, direct observations, and policy document reviews. Quantitative data were analysed using descriptive statistics and chi-square tests in SPSS version 26, while qualitative data were analysed thematically using NVivo 12. Mixed-methods integration occurred at the design, analysis, and interpretation stages. Medical waste generation ranged from 0.116 to 0.561 kg/bed/day across facilities. Only 61% of respondents correctly identified that waste segregation should occur at source, while only 33.3% correctly identified pharmaceutical waste colour-coding requirements. Storage durations ranged from 5–10 days, substantially exceeding WHO recommendations of 24–48 h. Pharmaceutical waste was frequently mixed with general and infectious waste streams, and no facility reported specialised treatment for antimicrobial-containing waste. Knowledge of waste categorisation varied substantially by professional category, with the highest accuracy among doctors and laboratory scientists and the lowest among domestic workers/cleaners (χ^2^ = 1344.00, df = 20, *p* < 0.001). Because approximately 70% of contingency table cells had expected counts below five, this association is reported as an exploratory, descriptive pattern rather than a robust inferential result throughout this manuscript. Policy analysis revealed limited integration of AMR considerations into healthcare waste management regulations and weak institutional coordination. The findings suggest that current medical waste management practices in Lagos may create conditions that facilitate environmental dissemination of antimicrobial residues and resistant microorganisms. Strengthened segregation, improved storage and treatment systems, environmental monitoring, and integrated governance approaches are required. Future studies should directly measure antimicrobial residues, resistant bacteria, and resistance genes in environmental media to validate the pathways identified in this study.

**Supplementary Information:**

The online version contains supplementary material available at https://doi.org/10.1186/s12889-026-28840-w.

## Introduction

### Background and significance

Antimicrobial resistance threatens to cause 10 million deaths annually by 2050 if current trends continue [26]. While clinical antimicrobial use receives considerable attention as an AMR driver, the role of inadequate medical waste management remains critically underexamined, particularly in low- and middle-income countries with deficient waste infrastructure [20]. Healthcare facilities generate substantial waste containing antimicrobial residues, resistant bacteria, and mobile genetic elements – materials that, when improperly managed, contribute to AMR dissemination in the environment [37].

Nigeria exemplifies the convergence of factors amplifying AMR risks through medical waste pathways. The country’s healthcare system generates over 500,000 tons of medical waste annually [10], yet lacks comprehensive treatment and disposal infrastructure. Lagos State, with 20 + million inhabitants and serving as Nigeria’s commercial centre, faces particularly acute challenges as concentrated healthcare facilities, rapid urbanisation, and inadequate waste management systems create conditions that may facilitate AMR propagation [28].

The medical waste–AMR nexus operates through multiple mechanisms. Pharmaceutical waste containing active antimicrobial compounds creates selective pressure for resistance development in environmental bacteria [12], infectious waste from antimicrobial-treated patients harbours resistant bacteria capable of horizontal gene transfer to environmental microorganisms [7], contaminated sharps and laboratory waste serve as transmission vectors to waste handlers and communities [4],and inadequate treatment facilities fail to inactivate antimicrobial compounds and resistant bacteria before disposal, allowing environmental persistence [32].

### Research gap and objectives

While existing literature documents medical waste management deficiencies in developing countries, the specific linkage between these practices and AMR propagation remains inadequately explored. Most studies address waste management or AMR in isolation, without examining mechanistic connections and pathways through which inadequate waste practices may contribute to resistance dissemination [20]. This study addresses this gap by systematically examining medical waste management in Lagos healthcare facilities through an AMR lens, identifying potential contamination pathways, and proposing integrated interventions.

### Objectives

(i) Assess current medical waste management practices with specific attention to antimicrobial-containing materials; (ii) Identify potential contamination pathways that may facilitate AMR dissemination; (iii) Evaluate policy and institutional frameworks governing medical waste management and their adequacy for addressing AMR risks; (iv) Develop evidence-based recommendations integrating AMR considerations into waste management policies and practices.

### Literature review

#### AMR and environmental dimensions

Antimicrobial resistance has evolved from clinical curiosity to global health emergency, with environmental compartments serving as critical reservoirs and transmission pathways for resistance genes and resistant bacteria [21]. Healthcare facilities contribute significantly through wastewater discharge, pharmaceutical waste disposal, and inadequate infectious waste treatment [8]. These environmental reservoirs facilitate horizontal gene transfer between bacterial species, creating opportunities for resistance dissemination beyond original hosts [25].

#### Medical waste as AMR vector

Medical waste represents a particularly concerning AMR vector due to concentrated antimicrobial agents, resistant microorganisms, and conducive environmental conditions throughout the waste management chain [15]. Infectious waste from antimicrobial-treated patients contains resistant bacteria,pharmaceutical waste includes active antimicrobials maintaining selective pressure; laboratory waste harbours cultured resistant organisms; and sharps contaminated with blood from treated patients serve as direct transmission vectors [27]. Mechanisms operate at multiple scales.

Antimicrobial compounds in pharmaceutical waste exert selective pressure on environmental bacterial populations [2], waste disposal sites create microenvironments with elevated bacterial density and stress conditions enhancing horizontal gene transfer [4],waste handlers, scavengers, and communities experience direct exposure through occupational contact and environmental contamination [10].

#### Medical waste management in developing countries

Studies across Asia, Africa, and Latin America document common deficiencies. These include inadequate segregation, mixing infectious and pharmaceutical wastes with general waste [20],insufficient treatment infrastructure [12],prolonged storage exceeding recommended timeframes [27],unsafe transport exposing waste handlers [29],and inadequate final disposal with untreated medical waste in open dumps [37]. These deficiencies reflect broader constraints including insufficient funding, limited technical capacity, weak regulatory enforcement, rapid healthcare expansion without corresponding waste planning, and competing priorities [4].

#### The Nigerian context

Nigeria’s policy framework includes the National Policy on Injection Safety and Healthcare Waste Management (2007) and National Healthcare Waste Management Plan, but implementation remains inconsistent [9]. Lagos has developed more robust infrastructure than most Nigerian regions, including the Lagos Waste Management Authority (LAWMA) medical waste unit, hydroclave treatment facilities, and private sector participation [1]. Despite these advances, studies document persistent deficiencies including inadequate segregation, treatment capacity constraints, prolonged storage, and limited verification of disposal practices [28]. Pharmaceutical waste handling receives insufficient attention in protocols and practices [3].

#### Environmental AMR pathways

Pathways through which inadequate treatment of medical waste may contribute to environmental AMR include the following. Wastewater discharge carrying antimicrobial residues and resistant bacteria [21],leachate from disposal sites contaminating groundwater and surface water [36],aerosol generation during incineration dispersing resistant bacteria [19],soil contamination creating resistance gene reservoirs [24],and direct exposure of waste handlers and communities [5].

#### Theoretical framework

This research adopts an integrated socio-ecological framework recognising AMR dissemination through medical waste as a complex system involving interactions across individual (healthcare worker practices), facility (infrastructure, management), policy (regulations, enforcement), community (exposure pathways), and environmental (AMR reservoirs) levels [6]. The framework incorporates environmental justice perspectives recognising vulnerable populations bear disproportionate risks [30] and political economy perspectives situating challenges within broader structural inequalities [35].

## Methodology

### Study Design and Setting

This concurrent mixed-methods study combined quantitative assessments with qualitative exploration of stakeholder perspectives, policy frameworks, and potential contamination pathways [16]. Conducted October 2021 – March 2022 in Lagos State’s Alimosho and Mushin Local Government Areas, the study selected Lagos due to its status as Nigeria’s largest city, concentration of healthcare facilities (25% of country’s private sector), relatively advanced waste infrastructure, and availability of comparative data [28].

### Sample selection and size

Healthcare facilities were selected using stratified purposive sampling to ensure representation across different levels of healthcare delivery. The final sample comprised seven facilities, including one federal tertiary hospital, one state secondary hospital, two primary healthcare centres, and three private hospitals.

For the quantitative component, 384 questionnaires were distributed to healthcare workers involved directly or indirectly in healthcare waste generation, handling, storage, transportation, treatment, and management activities. A total of 336 completed questionnaires were returned, representing a response rate of 87.5%.

Participants included doctors, nurses, laboratory scientists, cleaners, waste handlers, pharmacists, administrators, and other healthcare personnel. Because participants were recruited purposively from selected facilities rather than through probability sampling, findings should not be interpreted as statistically representative of all healthcare workers in Lagos State.

For the qualitative component, purposive and snowball sampling techniques were employed to identify key informants with relevant expertise and responsibilities. Forty-nine key informants participated in semi-structured interviews, comprising facility administrators (n = 7), infection prevention and control officers (n = 7), nurses (n = 12), laboratory personnel (n = 8), cleaners (n = 10), LAWMA officials (n = 3), and private sector waste operators (n = 2). Recruitment continued until thematic saturation was achieved.

### Data collection methods

#### Facility assessment

Structured assessments using direct waste measurement (weighing of waste containers at each generation point) and review of existing facility records (where available) documented waste generation rates and composition, segregation practices, storage infrastructure and duration, treatment capabilities, transport arrangements, disposal verification, and staff training. Each facility received 3–5 visits over the study period. A structured observational checklist adapted from WHO healthcare waste management assessment tools was used to systematically document practices at each facility [38].

#### Questionnaire survey

Structured questionnaires administered to healthcare workers comprised six sections: demographics (10 items), waste categorisation knowledge with AMR-specific items (15 items), segregation practices (12 items), storage and transport (8 items), training (6 items), and AMR-waste linkages awareness (10 items). The questionnaire was developed through literature review and consultation with environmental health experts.

The instrument was pilot tested among twenty healthcare workers prior to field implementation. Feedback obtained during pilot testing informed revisions to wording, structure, and response options. Content validity was assessed through expert review. However, formal psychometric validation and reliability testing were not undertaken and are acknowledged as study limitations.

Items were generated through a comprehensive review of existing literature on healthcare waste management assessment instruments and consultation with environmental health experts at Lagos State University of Science and Technology [40], and the questionnaire was modelled on the procedure used. The complete questionnaire is provided as Supplementary File S1.

#### Semi-structured interviews

In-depth interviews with key informants explored current practices, pharmaceutical/antimicrobial waste handling, AMR-waste awareness, policy frameworks, constraints, and improvement recommendations. Interviews lasted 30–75 min; with consent, were audio-recorded and transcribed verbatim. The semi-structured interview guide is provided as Supplementary File S2.

#### Observational data

Systematic observation documented practices at waste generation points, collection points, during transport, at transfer stations, and treatment/disposal facilities (where access permitted).

#### Document review

Analysis of national policies, facility records, regulatory documents, and international guidelines provided contextual understanding and identified policy–practice gaps.

### Data analysis

#### Quantitative

SPSS version 26 analysed questionnaire and facility data using descriptive statistics and chi-square tests examining associations between variables (significance *p* < 0.05). Effect sizes (Cramér’s V) were computed for significant chi-square results. All statistical results are reported with degrees of freedom.

#### Qualitative

Thematic analysis following Braun and Clarke’s framework coded interviews, observations, and documents using NVivo 12, an approach well suited to the exploratory, context-rich nature of qualitative health-care research [31]. Codebook developed iteratively organised codes into thematic categories. Inter-coder reliability (Cohen’s Kappa = 0.82) confirmed substantial agreement [17].

#### Integration

Mixed-methods integration occurred in design (concurrent data collection), analysis (qualitative data explaining quantitative patterns), and interpretation (synthesised findings addressing research questions from multiple perspectives).

### Ethical considerations

Ethical approval obtained from Lagos State Health Research Ethics Committee (LSHREC/10/06/2021) and individual facilities. All participants provided written informed consent. Facility anonymity maintained through alphabetical codes; individual anonymity through role-based identifiers. Data stored securely with limited access. Reporting of this study follows the STROBE checklist for cross-sectional and mixed-methods observational research, provided as Supplementary File S3.

## Results

### Facility and participant characteristics

Seven facilities served approximately 15,000 outpatients and 1,200 inpatients weekly. Among 336 questionnaire respondents, 31.4% were nurses (*n* = 106), 34.3% domestic workers/cleaners (*n* = 115), 11.4% doctors (*n* = 38), 10.5% laboratory scientists (*n* = 35), and 3.8% other professionals (*n* = 13). Mean age was 35.46 ± 1.66 years; 53.3% female; average employment duration 9.73 ± 6.91 years.

### Waste generation and composition

Total medical waste generation was 215.56 kg/day (range: 1.74–252.45 kg/day across facilities), averaging 0.181 kg/bed/day and varying by facility type (Table 1). Pharmaceutical waste – most relevant to AMR – was generated in all clinical areas, with the highest volumes from pharmacies (20.0%), medical wards (16.2%), and surgical wards (16.2%). However, pharmaceutical waste segregation was notably poor, with frequent mixing with general or infectious waste.

**Table 1:** Characteristics of surveyed healthcare facilities

Facility	Type	Beds	Waste (kg/day)	Waste (kg/bed/day)	Location (LGA)	Sector
A	Federal Teaching Hospital	450	252.45	0.561	Mushin	Public
B	State General Hospital	180	44.64	0.248	Mushin	Public
C	Primary Health Centre	20	2.68	0.134	Alimosho	Public
D	Primary Health Centre	15	1.74	0.116	Alimosho	Public
E	Private Hospital	50	9.90	0.198	Alimosho	Private
F	Private Hospital	30	8.01	0.267	Alimosho	Private
G	Private Hospital	40	8.14	0.204	Alimosho	Private

*LGA* Local Government Area, the principal sub-state administrative division in Nigeria. Mushin and Alimosho are the full names of the LGAs in which the surveyed facilities are located, not abbreviations

### Waste segregation practices

Only 61.0% of respondents correctly identified that segregation should occur at source; 39.0% indicated otherwise or did not know. This finding may reflect inadequate initial training and irregular refresher sessions, a pattern supported by qualitative data (see Sect. 4.8). When asked about actual locations, 52.2–90.0% reported source segregation (varying by facility), but 17.4–34.1% indicated segregation "outside the bin" or at collection points, suggesting remixing. Observational data confirmed frequent mixing. At Facilities C and D, pharmaceutical waste including antimicrobials was observed disposed in general black bags or mixed with infectious waste.

Colour-coding knowledge varied, with 58.1% correctly identifying black for general waste, but only 33.3% correctly identifying brown for pharmaceutical waste – critical for antimicrobial management.

### Knowledge of waste categorisation

Respondents demonstrated varying levels of knowledge regarding healthcare waste categorisation.

Approximately 69.5% correctly identified paper, food remnants, and uncontaminated plastics as general waste. Similar proportions correctly categorised contaminated cotton wool, swabs, and gloves as infectious waste. However, significant levels of misclassification were observed for waste streams relevant to antimicrobial resistance and infection control.

Only 58.1% correctly identified pathological waste, while substantial proportions misclassified pathological materials as either infectious or general waste. Misclassification of pharmaceutical waste was particularly common.

Knowledge levels varied substantially by professional category (Table 2), with a chi-square test indicating a statistically significant association between professional category (5 groups) and waste categorisation accuracy (χ^2^ = 1344.00, df = 20, *p* < 0.001). The degrees of freedom (df = 20) correspond to a 5 × 6 contingency table [(5 − 1) × (6 − 1) = 20], reflecting five professional categories and six response levels for the categorisation item; this dimensionality was verified against the original SPSS crosstabulation output prior to this submission. Because approximately 70% of contingency table cells had expected frequencies below five, chi-square assumptions were violated and the test statistic, along with the associated effect size (Cramér’s V ≈ 1.00), is likely inflated. Cramér’s V is therefore reported here purely to describe the approximate magnitude of the observed association and should not be interpreted as a precise or reliable effect size given the violated assumptions. Accordingly, this result is reported here, and interpreted throughout the manuscript, as an exploratory and descriptive pattern rather than a robust inferential finding, and should not be taken as evidence of a near-perfect association between the two variables.

**Table 2:** Knowledge and awareness indicators by professional category

Indicator	Doctors (*n* = 38)	Lab Scientists (*n* = 35)	Nurses (*n* = 106)	Domestic Workers (*n* = 115)	Overall (*N* = 336)
AMR awareness (%)	73.7	68.6	45.3	18.3	42.6
Training received (%)	94.7	85.0	89.5	52.3	74.3
Correct segregation knowledge (%)	78.9	74.3	71.7	42.6	61.0
Pharma colour-coding (%)	55.0	48.0	35.0	12.0	33.3
AMR content recalled in training (%)	31.6	28.6	18.9	5.2	18.4
Pharma waste procedures aware (%)	89.5	82.9	79.2	58.3	74.3
Needlestick injury reported (%)	10.5	14.3	18.9	35.7	23.8

Knowledge levels were highest among nurses and laboratory scientists, followed by doctors, while domestic workers and cleaners demonstrated the lowest levels of categorisation accuracy. This disparity is noteworthy because cleaners are responsible for handling a substantial proportion of healthcare waste throughout the waste management chain.

Interview participants consistently attributed these knowledge gaps to limited access to training opportunities and insufficient inclusion of cleaners and waste handlers in formal waste management programmes.

### Pharmaceutical and antimicrobial waste management

#### Identification and segregation

Only 74.3% reported awareness of specific pharmaceutical waste procedures, leaving over 25% without guidance. Observational data revealed inconsistent segregation.

#### AMR awareness

Only 42.6% of respondents were aware that improper antimicrobial disposal contributes to environmental AMR. Awareness was highest among doctors (73.7%) and laboratory scientists (68.6%), moderate among nurses (45.3%), and lowest among domestic workers (18.3%). Infection control officers acknowledged AMR concerns but reported training rarely addressed AMR–waste connections explicitly.

Table 2 summarises the knowledge and awareness indicators stratified by professional category, highlighting the steep gradient from clinical staff to waste handlers across all measured dimensions.

#### Treatment and disposal

No facility reported specialised on-site treatment for pharmaceutical waste. All outsourced to LAWMA-accredited private sector participants (PSPs), with pharmaceutical waste treated through the same hydroclave process as other medical waste. However, hydroclave technology may not effectively degrade chemical antimicrobial compounds despite sterilising biological materials [13]. Post-treatment waste fate remained unclear.

### Storage practices and duration

WHO guidelines recommend storage not exceeding 24–48 h [39]. Results revealed widespread non-compliance.

**Fig. 1:**
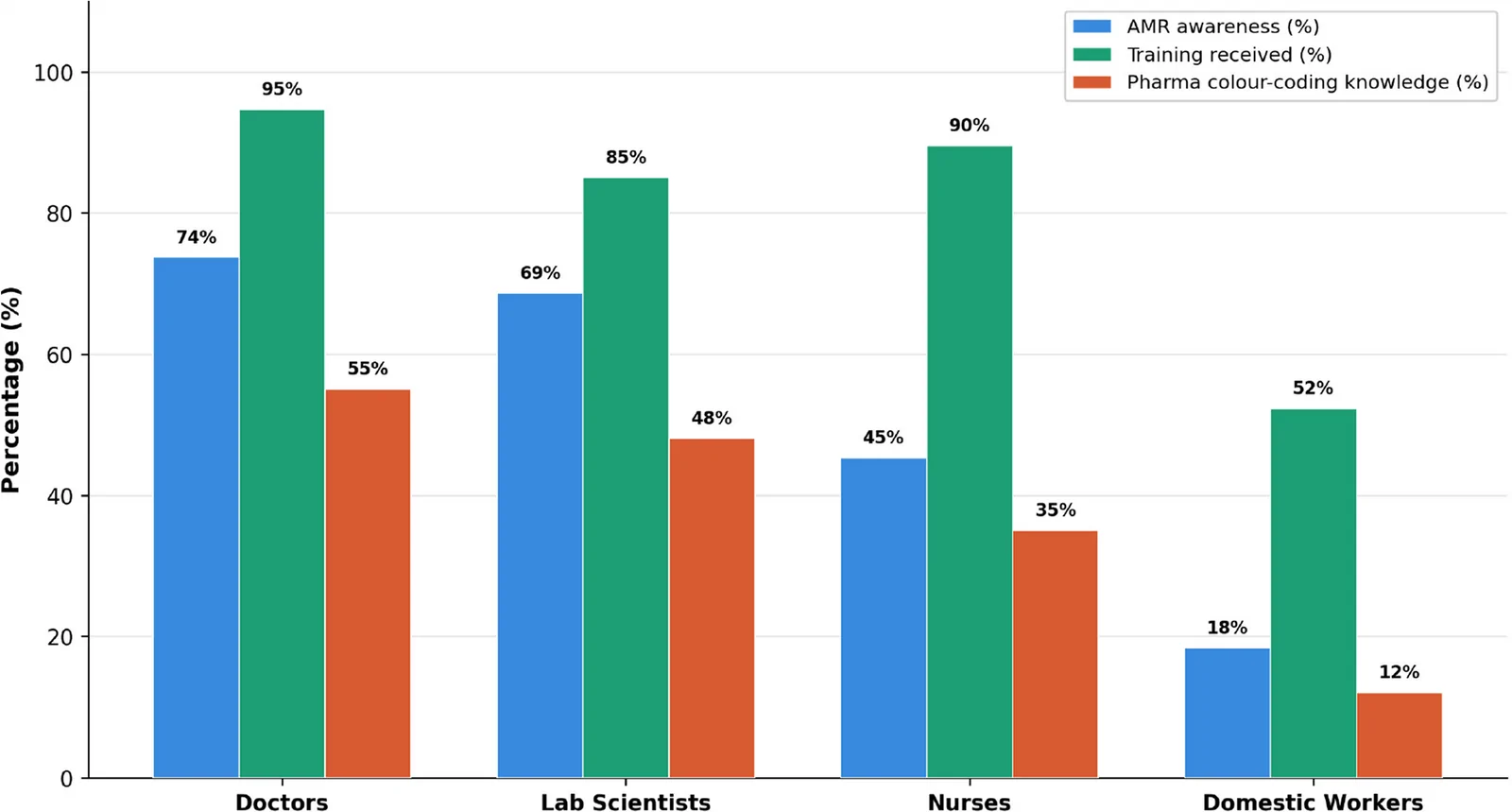
AMR awareness, training received, and pharmaceutical colour-coding knowledge across professional categories

Figure 1 visually illustrates this profession-stratified disparity across AMR awareness, training rates, and pharmaceutical colour-coding knowledge.

### On-site storage

At Facility A, bedside bins were collected four to five times daily, suggesting 2–6 h storage at generation points. However, after ward collection, waste stored in temporary holding areas (sluice rooms) added several hours. At Facilities C and D, sluice room bins were collected when full rather than on schedule, with cleaners reporting intervals of one to two days.

Table 3 compares observed storage durations at each stage against WHO-recommended limits, showing non-compliance particularly at the central storage and collection stages.

**Table 3:** Comparison of Observed Storage Durations with WHO Guidelines

Storage Point	Observed Duration	WHO Guideline	Conditions	Compliance Status
Bedside bins	2–6 h	24–48 h	Ward temperature	Compliant
Sluice rooms	Several hours–2 days	24–48 h	Unrefrigerated	Variable
Central storage	5–10 days	24–48 h	27–32 °C, 60–85% RH	Non-compliant
LAWMA collection	Weekly (irregular)	Within 48 h	Open vehicles	Non-compliant

### Central storage

Most significant violations occurred at central storage. All seven facilities reported LAWMA PSP collection approximately weekly, with actual intervals of 5–10 days depending on volume and schedules. Storage areas were typically unrefrigerated (ambient 27–32 °C), exposed to direct sunlight, not climate-controlled, with high humidity (60–85%), and variably secured. These conditions may be conducive to bacterial growth, though the extent of AMR amplification under these storage conditions was not directly measured in this study.

### Waste transportation and treatment

#### Internal transport

Wheelbarrows and trolleys were most common (68.2%), but manual carrying was also observed. Notably, 26.8% indicated transport through clinical areas used by patients, creating exposure pathways.

#### External transport

Collection vehicles bore LAWMA markings but were often standard pickups with open beds or partially covered containers. No specialised containment was observed for pharmaceutical waste. Collection frequency averaged weekly, though irregular.

#### Treatment

LAWMA operates a centralised hydroclave treatment facility. Concerns emerged regarding limited capacity relative to Lagos’s waste generation, minimal treatment verification, and unclear post-treatment disposal pathways.

#### Final disposal

Ultimate waste fate remained the opaquest element. Facility staff uniformly reported not knowing what happens after LAWMA collection. LAWMA officials stated that after treatment, waste goes through normal disposal channels – predominantly meaning open dumpsites or semi-controlled landfills where waste pickers commonly scavenge. The possibility that chemical antimicrobial compounds may persist in treated waste and enter disposal environments warrants further investigation through environmental sampling studies.

### Staff training and knowledge gaps

While 74.3% reported receiving some training, the quality, content, and frequency varied widely. Formal training was most common at public facilities (85–95%) versus private facilities (60–75%) and primary health centres (25–45%). Training was least common among cleaners, with only 52.3% reporting any training versus 89.5% of nurses and 94.7% of doctors. Training focused primarily on basic segregation, colour-coding, and occupational safety. AMR–waste linkages were rarely addressed, with only 18.4% of trained respondents recalling AMR content and 12.7% recalling pharmaceutical waste content specifically. Training frequency was inadequate, consisting of initial orientation with irregular refreshers.

### Environmental contamination pathways

Direct environmental sampling for antimicrobial residues and resistant bacteria was not conducted in this study. However, observational data and stakeholder accounts identified multiple potential contamination pathways that warrant further investigation through environmental monitoring studies.

The five pathways described below differ in their evidentiary basis. Some rest directly on practices this study observed or on facility and LAWMA staff accounts (e.g. untreated liquid waste discharge, unsecured storage, transport spillage, and waste-picker access to disposal sites); these are treated here as observed practices with a plausible, literature-supported contamination mechanism. Others – principally whether antimicrobial residues persist through hydroclave treatment and subsequently contaminate soil, groundwater, or surface water at disposal sites – are hypothesised mechanisms consistent with the treatment and disposal practices observed, but they were not directly measured and require confirmation through environmental sampling. Table 4 summarises each pathway, its evidentiary basis, and its priority for future environmental sampling.

**Table 4:** Environmental contamination pathways, evidentiary basis, and sampling priority

Pathway	Evidentiary Basis	Classification	Sampling Priority
1. Wastewater discharge	Observed (untreated discharge into sewage at all facilities)	Observed practice	High
2. Storage-site practices	Observed (leakage, rain infiltration, lack of impermeable flooring)	Observed practice	High
3. Transport spillage	Observed (visible spillage during loading and movement)	Observed practice	Moderate
4. Waste-picker exposure	Stakeholder report (LAWMA officials; observed access at disposal sites)	Observed/stakeholder-reported	High
5. Occupational exposure	Observed/self-reported (needlestick injuries, glove-use gaps)	Observed practice	Moderate
6. Post-treatment residue persistence and disposal-site leaching	Not directly measured; inferred from hydroclave technology limitations and open-dumpsite disposal	Hypothesised	High

#### Pathway 1—Wastewater discharge

All facilities disposed liquid waste (laboratory chemicals, disinfectants, pharmaceutical liquids) into sewage without treatment, which may deliver antimicrobial residues into wastewater systems.

#### Pathway 2—Storage site

Practices**:** Storage sites showed evidence of poor practice. Waste bags leaked onto floors and ground; rain infiltration mobilised contaminants; facilities lacking impermeable flooring allowed potential soil contamination; storm drains near storage provided pathways to stormwater systems.

#### Pathway 3—Transport spillage

Transport created spillage potential, with bags catching and breaking during facility movement, visible spillage during loading onto collection vehicles, and open transport vehicles allowing potential aerosol generation.

#### Pathway 4—Waste picker exposure

Despite security attempts, waste picking occurred, particularly at less-secure sites and final disposal locations. LAWMA officials acknowledged pickers access disposal sites.

**Fig. 2:**
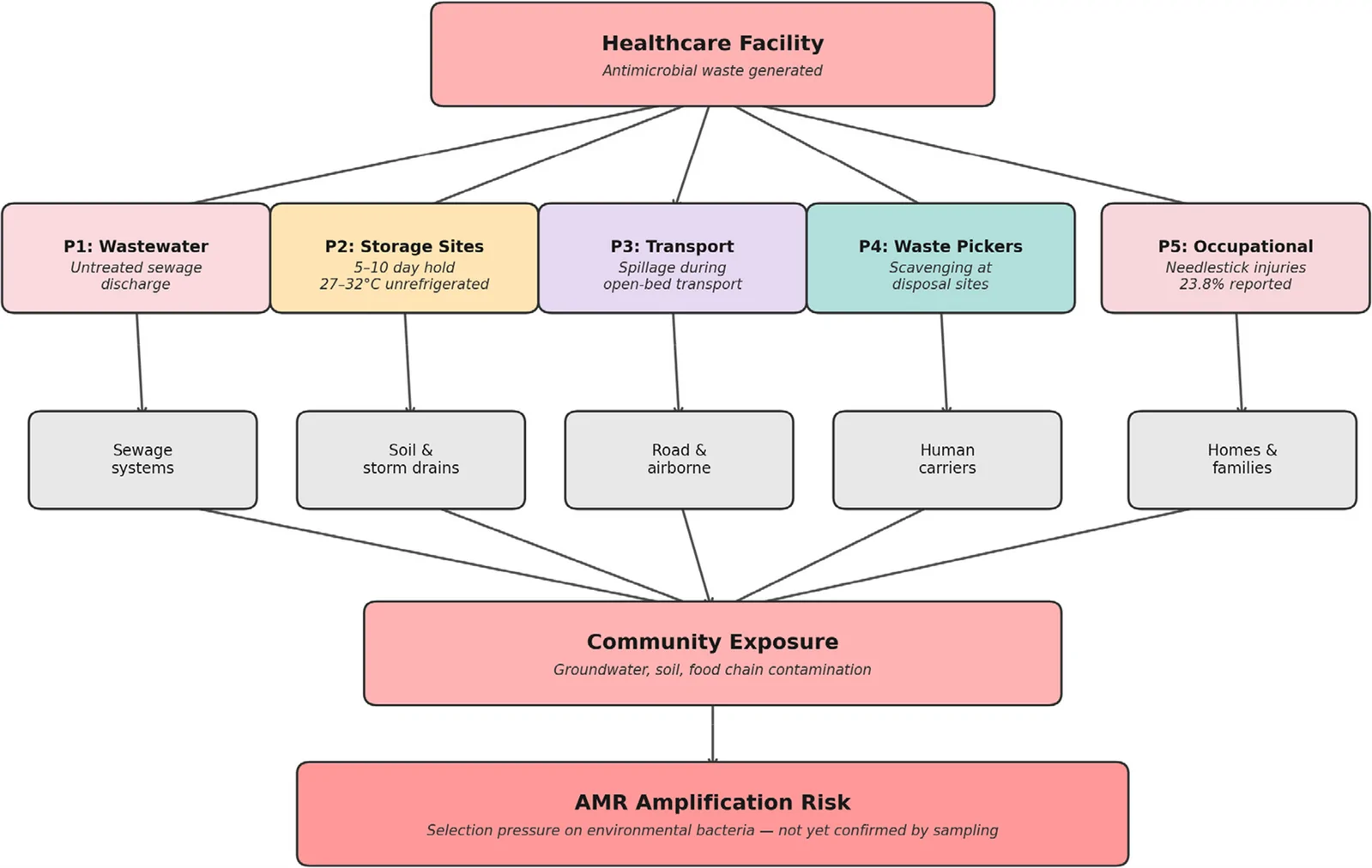
Environmental contamination pathways from healthcare waste to community AMR exposure

Figure 2 maps these five contamination pathways, illustrating how antimicrobial waste progresses from healthcare facilities through intermediate environments to community-level exposure and potential AMR amplification.

#### Pathway 5—occupational exposure

Healthcare workers, especially cleaners, experienced direct exposure through needle stick injuries (23.8% reported at some point), contact despite glove use, inhalation of aerosols, and clothing/footwear contamination potentially transferring pathogens to homes and communities.

### Policy and institutional framework

#### National policy gaps

National policies provide general frameworks but have significant AMR limitations. Pharmaceutical waste receives limited attention with no specialised antimicrobial requirements; AMR is not explicitly mentioned as a waste management rationale; environmental monitoring requirements are absent; enforcement mechanisms are weak with insufficient penalties; and there is minimal integration with antimicrobial stewardship frameworks. Applying Kingdon’s [22] multiple streams framework, the problem stream (AMR risks from medical waste) and policy stream (available technical solutions) have not converged with the political stream (governmental will and priority-setting) to open a policy window for reform. AMR remains framed primarily as a clinical prescribing issue rather than an environmental and waste management challenge, limiting cross-sectoral policy development.

#### Institutional fragmentation

Responsibilities are distributed across multiple agencies with unclear coordination, contributing to oversight gaps, inconsistent guidance, resource inefficiencies, and accountability diffusion. This fragmentation reflects what Grindle [14] describes as an implementation gap, where policy design fails to account for the institutional complexity required for effective execution. The interests of different agencies (Federal Ministry of Health, Federal Ministry of Environment, NESREA, State agencies) may not align, and no single entity has both the mandate and capacity to coordinate AMR-oriented waste management.

#### Implementation deficits

Capacity constraints limit inspection abilities; financial limitations prevent infrastructure investments; technical gaps mean personnel lack understanding; and political will varies with medical waste as a lower priority. Enforcement deficits persist because the political costs of imposing regulations on under-resourced healthcare facilities are perceived as high, while the diffuse and long-term nature of AMR harms reduces political urgency.

#### LAWMA/PSP model

The outsourcing approach has advantages but limitations for AMR control. Accountability is diluted with facilities having limited oversight after collection; transparency is limited with PSPs treating details as proprietary; cost pressures may incentivise corner-cutting; and AMR-specific considerations are absent from contracts and monitoring.

#### Resource and capacity constraints

All facilities reported inadequate budgets for waste management. Private facilities particularly struggled, with waste management fees representing significant operating costs. Infrastructure deficits included inadequate storage facilities lacking climate control, insufficient colour-coded containers, limited transport equipment, and erratic personal protective equipment supplies. Staff shortages affected waste management, with suboptimal cleaner-to-bed ratios and infection control officers burdened with competing responsibilities.

## Discussion

### Medical waste management practices and AMR risk in Lagos

This study provides evidence suggesting that inadequate medical waste management in Lagos creates multiple potential pathways for AMR dissemination. The finding that only 61% correctly identify segregation procedures, combined with frequent mixing of pharmaceutical waste with general streams, creates conditions where antimicrobial-containing materials may receive inadequate treatment. Across surveyed facilities, the 215 kg/day total waste generation with 5–8% pharmaceutical waste suggests meaningful quantities of antimicrobial-containing materials are managed through inadequate pathways. While citywide extrapolation from seven facilities is highly uncertain, these findings indicate the scale of the challenge [21, 37].

These waste generation rates (0.181 kg/bed/day average) are broadly consistent with findings from other Nigerian studies. Awodele et al. [3] reported comparable rates in Lagos hospitals, while Olukanni et al. [28] documented similar patterns of pharmaceutical waste mixing with general waste streams across Nigerian healthcare facilities. However, the segregation compliance rate of 61% in the present study is lower than the 72% reported by Chisholm et al. [4] in a multi-country African review, suggesting particularly acute challenges in the Lagos context.

### Critical control point failures

#### Point of generation

Inadequate segregation represents the most fundamental failure. Only 33.3% correctly identify pharmaceutical waste colour-coding; observations showed routine mixing. Qualitative data illuminates the drivers of this pattern. Cleaners, who handle the majority of physical waste, reported that training was rare and focused on basic infection prevention rather than waste categorisation. As one cleaner at Facility D explained, they were uncertain about different waste types because no one had provided specific guidance on pharmaceutical waste. Infrastructure constraints compounded knowledge deficits, with multiple facilities reporting recurrent stockouts of colour-coded containers, forcing workers to use available bags regardless of waste type.

Similar patterns of inadequate pharmaceutical waste segregation have been documented across developing countries. Khan et al. [20] found that pharmaceutical waste segregation was consistently the weakest category across Asian healthcare facilities. Olaniyi et al. [27] reported comparable deficiencies in South African rural hospitals, where colour-coding knowledge for pharmaceutical waste was lowest among all waste categories.

#### Storage duration

Storage routinely exceeding 24–48 h limits, with actual durations of 5–10 days, creates extended periods during which bacterial proliferation may occur. The tropical Lagos conditions (27–32 °C, high humidity) in unrefrigerated facilities may exacerbate this risk, though the actual extent of AMR amplification during storage was not measured in this study and warrants future investigation.

These storage durations substantially exceed those reported by Padmanabhan and Barik [29], who found 2–3 day average storage in Indian hospitals, and by Kenny and Priyadarshini [18], who noted 3–5 day storage in comparable LMIC settings. The longer durations in Lagos facilities likely reflect logistical challenges specific to the LAWMA/PSP collection model.

#### Treatment adequacy

Reliance on hydroclave for all medical waste raises AMR adequacy questions. While hydroclave effectively sterilises biological materials (killing resistant bacteria), it does not chemically degrade antimicrobial compounds that may persist in treated waste [13, 18].

#### Final disposal

The opacity surrounding final disposal is most concerning. LAWMA’s statement that treated waste goes through normal disposal channels suggests disposal in Lagos’s municipal dumpsites where waste picking occurs and environmental controls are minimal. The possibility that antimicrobial residues may persist in treated waste and leach into soil and groundwater at disposal sites warrants investigation through environmental sampling studies [36].

#### Occupational exposure and equity considerations

Waste handlers, predominantly low-income cleaners, experience the greatest occupational exposure but receive the least training (52.3% vs. 89–95% for professional staff), reflecting systematic marginalisation. These workers face daily exposure through direct contact, needle sticks (23.8% reported), and aerosol inhalation, often with inadequate personal protective equipment. Waste pickers at disposal sites represent another vulnerable population, experiencing exposure without even the limited protections afforded to formal healthcare workers. Cook and Velis [5] and Schenck et al. [33] have documented comparable exposure risks among waste pickers in other settings.

These findings highlight the need for future research that includes environmental sampling near waste storage and disposal sites to determine whether the observed poor practices translate into measurable environmental contamination affecting nearby communities [27, 30].

#### Policy and governance challenges

Nigeria’s National Healthcare Waste Management Plan lacks AMR-specific provisions, reflecting broader disconnect between waste management and AMR policy domains. This siloed approach means waste policies focus on infection prevention and occupational safety without considering AMR implications, while AMR policies focus on clinical use and surveillance without addressing waste management pathways [9]. Drawing on Kingdon’s [22] multiple streams model, this disconnect reflects the failure of the AMR-waste problem to be coupled with viable policy solutions at a moment of political receptivity. The cross-cutting nature of the issue – spanning health, environment, and municipal services – means no single policy entrepreneur has championed it.

Institutional fragmentation compounds these challenges. Responsibilities distributed across multiple agencies create coordination challenges and accountability diffusion [10]. Implementation deficits are sustained by resource constraints, technical capacity gaps, and variable political will [23, 34].

### Proposed framework for strengthening healthcare waste management and AMR control

Based on the findings, an integrated framework is proposed comprising five mutually reinforcing components:

- Component 1: Enhanced segregation systems
  Strengthening segregation practices through improved infrastructure, colour-coded containers, supervision, and compliance monitoring.
- Component 2: Pharmaceutical waste management
  Establishing dedicated systems for segregation, collection, treatment, and disposal of antimicrobial-containing waste streams.
- Component 3: Safe storage and transportation
  Improving storage infrastructure, reducing storage durations, strengthening transportation systems, and enhancing occupational safety measures.
- Component 4: Environmental monitoring and surveillance
  Implementing surveillance programmes capable of measuring antimicrobial residues, resistant bacteria, and resistance genes in wastewater, soils, and disposal environments.
- Component 5: Integrated governance and one health coordination
  Promoting coordination among health, environmental, waste management, and regulatory agencies while integrating healthcare waste management into broader AMR strategies.

This five-component framework is intended to complement, rather than duplicate, existing AMR policy instruments. It operationalises relevant commitments of the WHO Global Action Plan on Antimicrobial Resistance – particularly those addressing infection prevention, environmental hygiene, and sanitation – at the level of routine healthcare waste handling. It similarly supports implementation of Nigeria’s National Action Plan for Antimicrobial Resistance [11] by providing a practical, facility-level mechanism through which the Plan’s environmental and One Health objectives can be advanced in Lagos State and comparable urban settings.

### Implementation priorities

Given resource constraints, a phased implementation approach is recommended.

Immediate priorities (0–12 Months)

- Mandatory healthcare waste management refresher training.
- Inclusion of AMR modules in staff training programmes.
- Provision of pharmaceutical waste containers.
- Routine segregation audits and compliance monitoring.
- Enhanced supervision of waste handling activities.

Medium-Term Priorities (1–3 Years)

- Upgrading storage infrastructure.
- Increasing waste collection frequency.
- Standardising pharmaceutical waste treatment procedures.
- Expanding regulatory inspections and enforcement activities.
- Improving occupational health protection systems.

Long-Term Priorities (3–5 Years)

- Establishing environmental AMR surveillance programmes.
- Developing national healthcare waste–AMR databases.
- Investing in advanced treatment technologies.
- Integrating healthcare waste indicators into national AMR monitoring frameworks.
- Strengthening One Health governance structures.

Prioritisation of interventions according to implementation timelines may improve feasibility and sustainability while allowing gradual strengthening of healthcare waste management systems.

### Strengths and limitations

#### Strengths of the study

This study makes several important contributions to the growing body of literature on healthcare waste management and antimicrobial resistance (AMR) in low- and middle-income countries. First, it is among the few studies in Nigeria to explicitly examine healthcare waste management through an antimicrobial resistance lens, thereby addressing an important gap in existing research. While previous investigations have focused primarily on waste generation, segregation, treatment, and disposal practices, this study extends the discussion by exploring how healthcare waste management systems may contribute to environmental dissemination of antimicrobial residues, resistant microorganisms, and antimicrobial resistance genes.

Second, the use of a concurrent mixed-methods design enabled a comprehensive assessment of healthcare waste management practices. The integration of quantitative surveys, facility assessments, direct observations, semi-structured interviews, and policy document reviews facilitated methodological triangulation and strengthened the validity of findings. Combining these multiple sources of evidence allowed the study to move beyond simple descriptions of waste management practices and provide a more nuanced understanding of the institutional, behavioural, environmental, and governance factors influencing implementation.

Third, the inclusion of multiple categories of healthcare facilities—including tertiary, secondary, primary, and private healthcare institutions—provided insights into variations in healthcare waste management practices across different levels of service delivery. This enhanced the contextual relevance of findings and enabled identification of facility-specific challenges and opportunities for improvement.

Fourth, the application of a socio-ecological framework provided a structured approach for understanding the interactions among individual knowledge and behaviours, institutional systems, policy environments, community exposure pathways, and environmental risks. The framework facilitated interpretation of findings across multiple levels and supported the development of integrated recommendations for policy and practice.

Finally, the study contributes to ongoing discussions regarding the environmental dimensions of antimicrobial resistance and supports the growing recognition of healthcare waste management as an important component of One Health approaches to AMR prevention and control.

#### Limitations of the study

Several limitations should be considered when interpreting the findings.

First, the study employed purposive sampling for both facility selection and participant recruitment. Although this approach was appropriate for obtaining information-rich cases and capturing diverse perspectives, it limits the statistical generalisability of findings beyond the participating facilities. Consequently, results should be interpreted as representative of the study settings rather than all healthcare facilities in Lagos State or Nigeria.

Second, the study relied partly on self-reported data obtained through questionnaires and interviews. As with all self-reported measures, responses may have been influenced by recall bias, reporting bias, or social desirability bias. To minimise these effects, survey findings were triangulated with direct observations, facility assessments, and documentary evidence.

Third, environmental sampling was beyond the scope of this research. Although the study identified several plausible pathways through which healthcare waste management practices may contribute to environmental dissemination of antimicrobial residues and resistant microorganisms, these pathways remain conceptual and inferential. Direct measurements of antimicrobial residues, resistant bacteria, and antimicrobial resistance genes in wastewater, soils, drainage systems, and disposal environments were not conducted. Consequently, the study cannot establish causal relationships between observed waste management practices and environmental AMR contamination.

Fourth, although the questionnaire was developed through literature review, expert consultation, and pilot testing, formal psychometric validation and reliability testing were not undertaken. The absence of measures such as Cronbach’s alpha represents a methodological limitation and should be considered when interpreting survey-based findings.

Fifth, a statistically significant association was observed between professional category and waste categorisation knowledge. However, approximately 70% of contingency table cells had expected frequencies below five, violating assumptions of the chi-square test. Although effect size estimates were reported, these findings should be interpreted as exploratory and descriptive rather than inferential.

Finally, the cross-sectional design provides a snapshot of healthcare waste management practices during the study period and does not permit assessment of temporal changes, intervention effects, or long-term trends. Future longitudinal studies would provide valuable insights into the effectiveness and sustainability of healthcare waste management improvements.

Despite these limitations, the study provides important baseline evidence regarding healthcare waste management practices, governance arrangements, and potential antimicrobial resistance pathways in Lagos State and contributes to the limited literature examining the healthcare waste–AMR nexus in sub-Saharan Africa.

#### Implications for research and practice

The findings highlight the need for future studies that directly measure antimicrobial residues, resistant bacteria, and resistance genes within healthcare waste systems and surrounding environments. Such research would provide empirical evidence regarding the relative importance of different contamination pathways and support the development of targeted interventions.

For policymakers and practitioners, the study underscores the importance of integrating healthcare waste management into broader antimicrobial resistance strategies. Strengthening segregation, pharmaceutical waste management, storage systems, treatment technologies, environmental surveillance, and governance arrangements may contribute not only to improved waste management outcomes but also to national and global efforts to combat antimicrobial resistance.

## Conclusion

This study assessed healthcare waste management practices across seven healthcare facilities in Lagos State, Nigeria, and examined their potential implications for antimicrobial resistance (AMR) dissemination. Using a mixed-methods approach that combined quantitative surveys, facility assessments, direct observations, stakeholder interviews, and policy analysis, the study provides a comprehensive assessment of healthcare waste management systems through an antimicrobial resistance perspective.

The findings reveal important deficiencies across multiple stages of the healthcare waste management chain. Significant challenges were identified in waste segregation, pharmaceutical waste management, storage practices, transportation systems, staff training, and regulatory implementation. Although many healthcare workers demonstrated awareness of basic healthcare waste management principles, substantial knowledge gaps remained regarding pharmaceutical waste categorisation, antimicrobial resistance pathways, and environmental contamination risks. Furthermore, observed practices frequently diverged from reported knowledge, highlighting the influence of institutional, infrastructural, and governance constraints on implementation outcomes.

The study identified several potential pathways through which healthcare waste management practices may contribute to environmental dissemination of antimicrobial residues, resistant microorganisms, and antimicrobial resistance genes. These pathways include wastewater discharge, storage-site contamination, transport-related spillage, occupational exposure, and community exposure associated with waste handling and disposal activities. While direct environmental measurements were beyond the scope of this research, the findings suggest that healthcare waste management should be recognised as an important component of broader antimicrobial resistance prevention and control strategies.

From a policy perspective, the study demonstrates that antimicrobial resistance considerations remain insufficiently integrated within existing healthcare waste management frameworks. Strengthening governance arrangements, improving institutional coordination, enhancing regulatory enforcement, and incorporating healthcare waste management into national AMR strategies will be essential for addressing emerging environmental resistance risks. The findings further support adoption of a One Health approach that recognises the interconnected relationships among human health, environmental systems, and antimicrobial resistance dynamics.

Based on the study findings, priority interventions include strengthening segregation systems, establishing dedicated pharmaceutical waste management procedures, improving storage and transportation infrastructure, expanding workforce training programmes, enhancing environmental monitoring, and developing integrated governance mechanisms capable of addressing healthcare waste management and antimicrobial resistance simultaneously.

Future research should focus on direct environmental sampling and laboratory analysis of antimicrobial residues, resistant bacteria, and antimicrobial resistance genes in wastewater, soils, drainage systems, treatment facilities, and disposal environments. Such investigations are urgently needed to validate the pathways identified in this study, quantify environmental AMR burdens, and inform evidence-based policy and intervention development.

In conclusion, healthcare waste management represents an important but frequently overlooked dimension of antimicrobial resistance prevention and control. Addressing deficiencies in healthcare waste systems offers an opportunity to strengthen environmental health protection, improve healthcare waste governance, and contribute to national and global efforts to combat antimicrobial resistance.

## Supplementary Information

Supplementary Material 1.

Supplementary Material 2.

Supplementary Material 3.

## Data Availability

The data supporting the findings of this study are available from the corresponding author upon request. Due to confidentiality agreements with participating healthcare facilities, raw data cannot be made publicly accessible.
