# Supplementary material for: Assessment of medical waste management practices and implications for antimicrobial resistance risk in urban Nigeria
Source: BMC Public Health. 2026 Aug 14;26:3307. doi: 10.1186/s12889-026-28840-w (PMC13753125; doi:10.1186/s12889-026-28840-w)
Supplement: Supplementary file 1 [file 12889_2026_28840_MOESM1_ESM.pdf]

Supplementary File S1

Questionnaire

Title: Healthcare Waste Management Practices and Antimicrobial Resistance (AMR) Risk Assessment Questionnaire

Section A: Demographic Information

1. Age: ________
2. Gender:
   - Male
   - Female
   - Prefer not to say
3. Professional Category:
   - Doctor
   - Nurse
   - Laboratory Scientist
   - Pharmacist
   - Cleaner/Domestic Worker
   - Waste Handler
   - Administrator
   - Other (Specify)
4. Highest Educational Qualification:
   - Primary
   - Secondary
   - Diploma
   - Bachelor's Degree
   - Master's Degree
   - Doctorate
   - Other
5. Years of Work Experience:
   - <1 year
   - 1–5 years
   - 6–10 years
     - 10 years
6. Department/Unit:

Section B: Knowledge of Healthcare Waste Categories

7. Which of the following is classified as general waste?
   - Food remnants
   - Blood-soaked cotton wool
   - Expired antibiotics
   - Used needles
8. Which colour code is used for pharmaceutical waste?
   - Black
   - Yellow

    - Brown
    - Red
9. Used syringes should be classified as:
    - General Waste
    - Infectious Waste
    - Sharps Waste
    - Pharmaceutical Waste
10. Expired antibiotics should be classified as:

- General Waste
- Pharmaceutical Waste
- Pathological Waste
- Infectious Waste

11. Human tissues and body parts belong to:

- Pathological Waste
- General Waste
- Pharmaceutical Waste
- Chemical Waste

12. Waste segregation should occur:

- At source
- During transportation
- At disposal site
- After collection

Section C: Waste Segregation Practices

13. Does your facility practice waste segregation?

- Yes
- No

14. Are colour-coded bins available in your work area?

- Always
- Sometimes
- Never

15. Are pharmaceutical wastes segregated separately?

- Always
- Sometimes
- Never

16. Have you observed mixing of medical waste streams?

- Frequently

- Occasionally
- Never

17. Are waste containers properly labelled?

- Yes
- No

18. Are segregation procedures monitored?

- Yes
- No

Section D: Storage and Transportation

19. How long is waste stored before collection?

- Less than 24 hours
- 24–48 hours
- 3–5 days
- More than 5 days

20. Is waste storage area secure?

- Yes
- No

21. Is storage area protected from rain and sunlight?

- Yes
- No

22. How is waste transported internally?

- Wheelbarrow
- Trolley
- Manual Carrying
- Other

23. Does transport pass through patient-care areas?

- Yes
- No

Section E: Training and Capacity Building

24. Have you received healthcare waste management training?

- Yes
- No

25. Frequency of training:

- Orientation only
- Annually
- Every 6 months
- Quarterly

26. Did the training include AMR-related content?

- Yes
- No

27. Did the training include pharmaceutical waste management?

- Yes
- No

28. Do you feel adequately trained?

- Yes
- No

Section F: Awareness of AMR and Waste Linkages

29. Have you heard of antimicrobial resistance?

- Yes
- No

30. Can improper disposal of antibiotics contribute to AMR?

- Yes
- No
- Not Sure

31. Can healthcare waste contaminate the environment?

- Yes
- No

32. Are waste handlers at risk of exposure to resistant organisms?

- Yes
- No

33. Should AMR considerations be included in healthcare waste management policies?

- Yes
- No

34. What improvements would you recommend?
