# Supplementary material for: Assessment of medical waste management practices and implications for antimicrobial resistance risk in urban Nigeria
Source: BMC Public Health. 2026 Aug 14;26:3307. doi: 10.1186/s12889-026-28840-w (PMC13753125; doi:10.1186/s12889-026-28840-w)
Supplement: Supplementary file 2 [file 12889_2026_28840_MOESM2_ESM.pdf]

Supplementary File S2

Semi-Structured Interview Guide

Interview Title

Healthcare Waste Management and Antimicrobial Resistance Risk in Lagos Healthcare Facilities

Participant Category

- Facility Administrator
- Infection Control Officer
- Doctor
- Nurse
- Laboratory Scientist
- Cleaner
- Waste Handler
- LAWMA Official
- PSP Operator

Opening Questions

1. Please describe your role within the facility.
2. How long have you worked in healthcare waste management activities?

Waste Management Practices

3. How is healthcare waste generated and managed in your facility?
4. Describe waste segregation procedures.
5. What challenges affect compliance?
6. How is pharmaceutical waste managed?
7. What happens to expired antibiotics?

Storage and Transportation

8. How long is waste stored before collection?
9. Are storage facilities adequate?
10. What challenges occur during transportation?

AMR Awareness

11. What is your understanding of antimicrobial resistance?
12. Have you received training on AMR?
13. Do you believe healthcare waste contributes to AMR dissemination?
14. How can pharmaceutical waste affect the environment?

Policy and Governance

15. Are you familiar with national healthcare waste policies?

16. How effective are current regulations?
17. What institutional challenges affect implementation?
18. Are there gaps relating to AMR?

Recommendations

19. What improvements are most urgently required?
20. How can government strengthen healthcare waste management?
21. What role should environmental monitoring play?
22. Any additional comments?
