# Supplementary material for: Assessment of medical waste management practices and implications for antimicrobial resistance risk in urban Nigeria
Source: BMC Public Health. 2026 Aug 14;26:3307. doi: 10.1186/s12889-026-28840-w (PMC13753125; doi:10.1186/s12889-026-28840-w)
Supplement: Supplementary file 3 [file 12889_2026_28840_MOESM3_ESM.pdf]

Supplementary File S3

STROBE Checklist

STROBE Item	Description	Page
Title/Abstract	Study design indicated	Abstract
Background	Scientific rationale	Introduction
Objectives	Clearly stated	Introduction
Study Design	Mixed-methods design described	Methods
Setting	Lagos facilities described	Methods
Participants	Sampling procedures described	Methods
Variables	Outcomes and exposures defined	Methods
Data Sources	Questionnaires, interviews, observations	Methods
Bias	Limitations discussed	Discussion
Study Size	Sample size reported	Methods
Quantitative Variables	Statistical methods reported	Methods
Statistical Methods	Chi-square and descriptive analyses	Methods
Participants	Numbers reported	Results
Descriptive Data	Demographics presented	Results
Outcome Data	Waste management findings reported	Results
Main Results	Quantitative and qualitative findings	Results
Discussion	Interpretation of findings	Discussion
Limitations	Explicitly discussed	Discussion
Generalisability	Addressed	Discussion
Funding	To be completed by authors	End of manuscript
